# A novel t (5; 17) (q35; q21) associated with t (8; 21) (q22; q22) in a patient with acute myeloid leukemia: case report and review of literature

**DOI:** 10.18632/genesandcancer.232

**Published:** 2023-06-28

**Authors:** Kmira Zahra, Wided Cherif, Gereisha Ahmed, Haifa Regaieg, Ben Sayed Nesrine, Monia Zaier, Wided Mootamri, Yosra Ben Youssef, Nejia Brahem, Halima Sennana, Abderrahim Khelif

**Affiliations:** ^1^Department of Clinical Hematology, Farhat Hached University Hospital-Sousse-Tunisia, Sousse 4081, Tunisia; ^2^Department of Cytology, Farhat Hached University Hospital-Sousse-Tunisia, Sousse 4081, Tunisia; ^3^Department of Cytogenetics, Farhat Hached University Hospital-Sousse-Tunisia, Sousse 4081, Tunisia

**Keywords:** acute myeloid leukemia, T (8; 21) (q22; q22), T (5; 17) (q35; q21), NPM1/RARA, allogenic stem cells transplantation

## Abstract

The t (8; 21) (q22; q22) with the resulting RUNX1- RUNX1T1 rearrangement is one of the most common cytogenetic abnormalities in acute myeloid leukemia (AML). It is associated with favorable prognosis. The t (5; 17) (q35; q21) is an uncommon translocation, fuses the gene for the nucleophosmin (NPM) to the retinoic acid receptor α(RARA) and was described essentially in acute promyelocytic leukemia (APL) variant. We present the case of a 19-year-old male patient who developed an AML with t (8; 21) (q22; q22) associated to t (5; 17) (q35; 21). Morphology and immunophenotype of the leukemic cells were compatible with AML. The patient received chemotherapy based on cytarabine and anthracycline without all-trans retinoic acid (ATRA) followed by allogenic stem cell transplantation in first remission. To the best of our knowledge, this is the first report of an association between a rare translocation t (5; 17) and t (8; 21) in AML. In this report, we will discuss the prognosis of this association as well as the treatment.

## INTRODUCTION

Acute myeloid leukemia (AML) is the most common acute leukemia in adults and counts for about 80% of all cases [1]. Cytogenetic analysis is the most important diagnostic tool for determining prognosis. In fact, numerous recurrent karyotype abnormalities have been discovered in AML. Commonly observed chromosomal abnormalities in AML are t (8; 21), t (15; 17), inv (16), which are associated with higher rates of complete remission and event free survival [2]. T (5; 17) in AML is exceptional, described in few cases in acute promyelocytic leukemia (APL) variant [3, 4].

Here we report an unusual association of t (5; 17) with t (8; 21) in AML and we try to discuss the prognosis of this association and then the treatment.

## CASE REPORT

**Figure 1:**
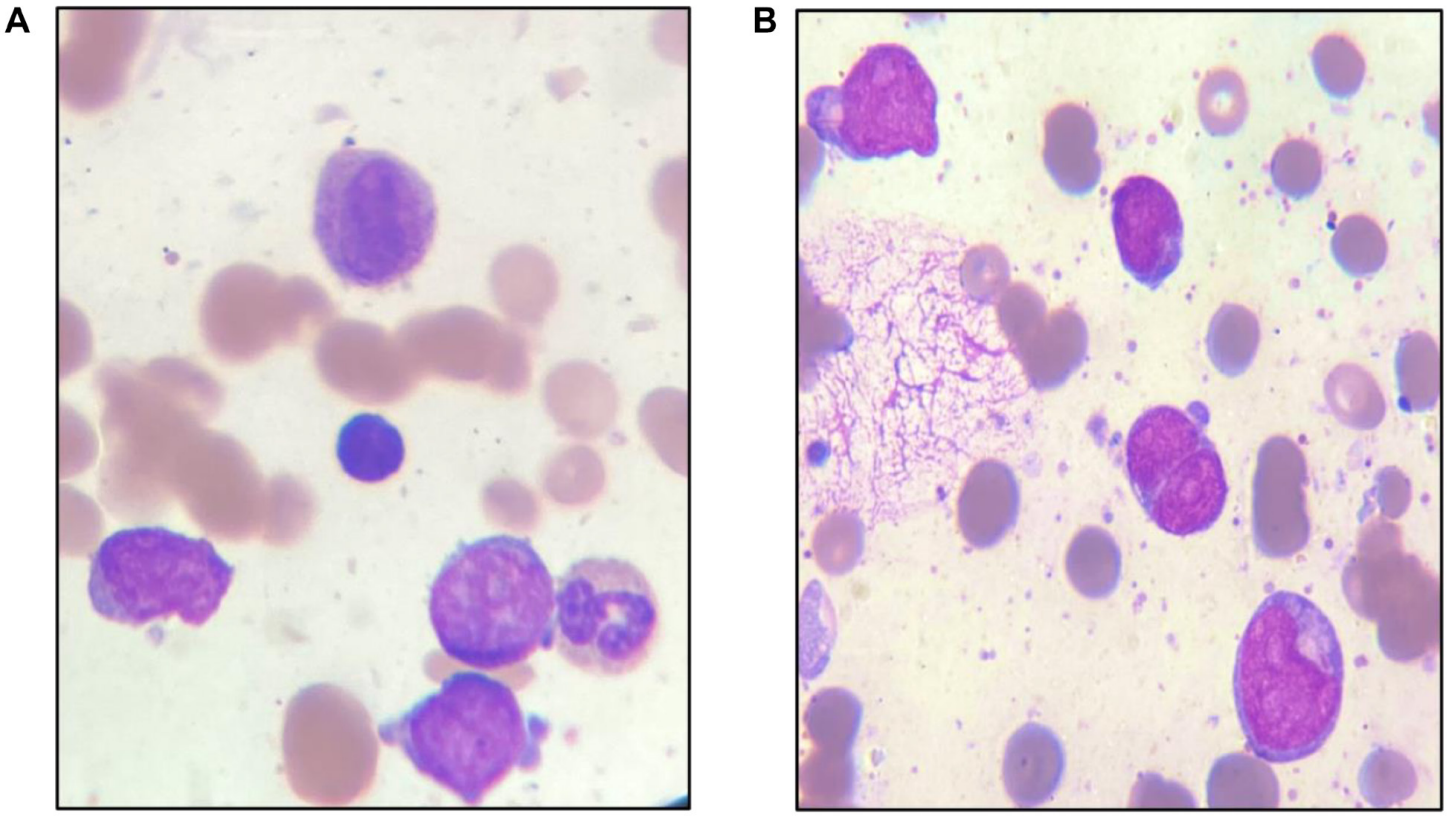
Note myeloblasts: Granular blast cells or with Auer bodies. (**A**) Peripheral Blood, (**B**) Bone marrow/wright stain × 100).

**Figure 2:**
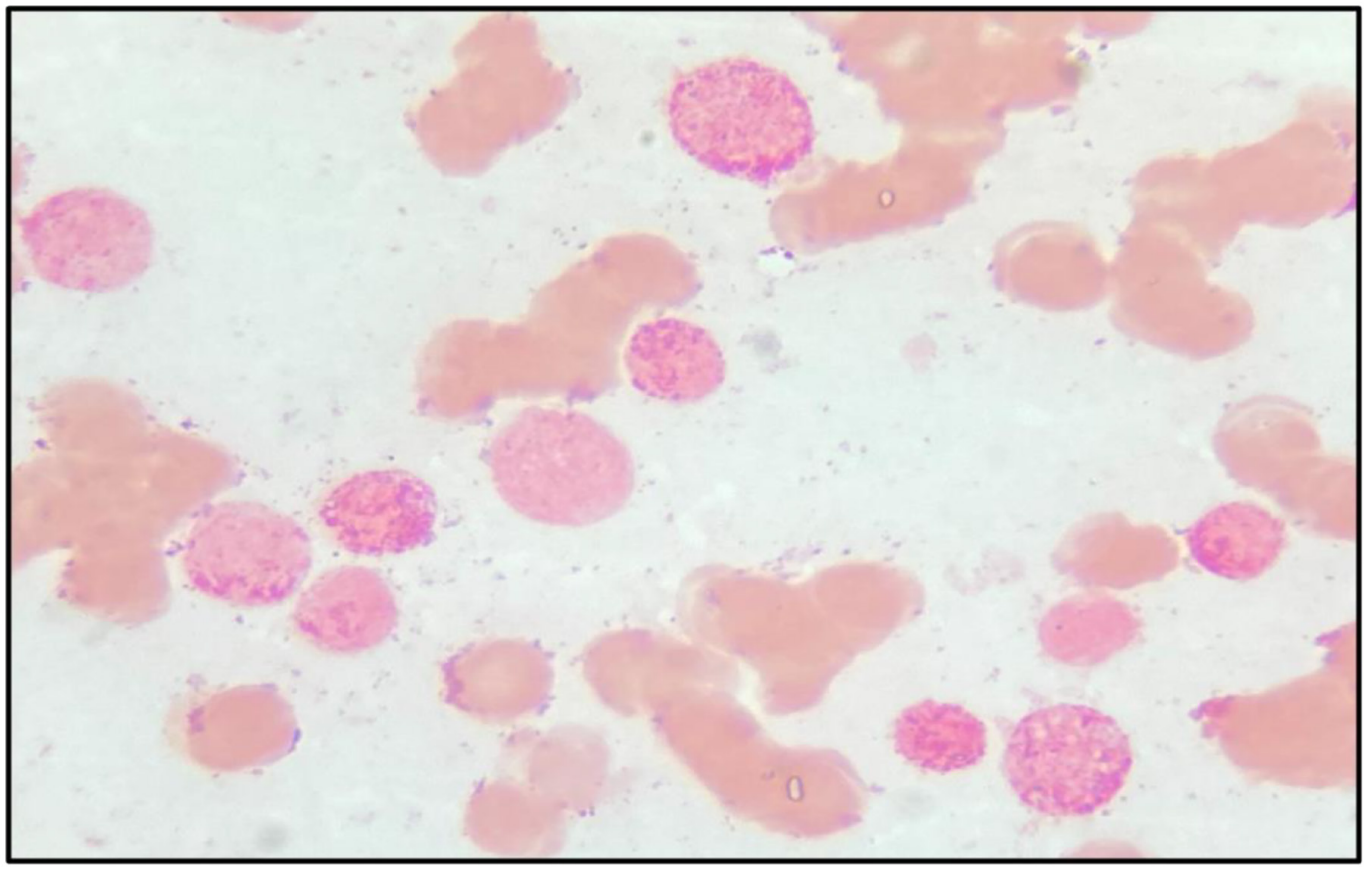
Bone marrow (Peroxydas/Pyronin stain × 100): blast cells strongly peroxydase+.

**Figure 3:**
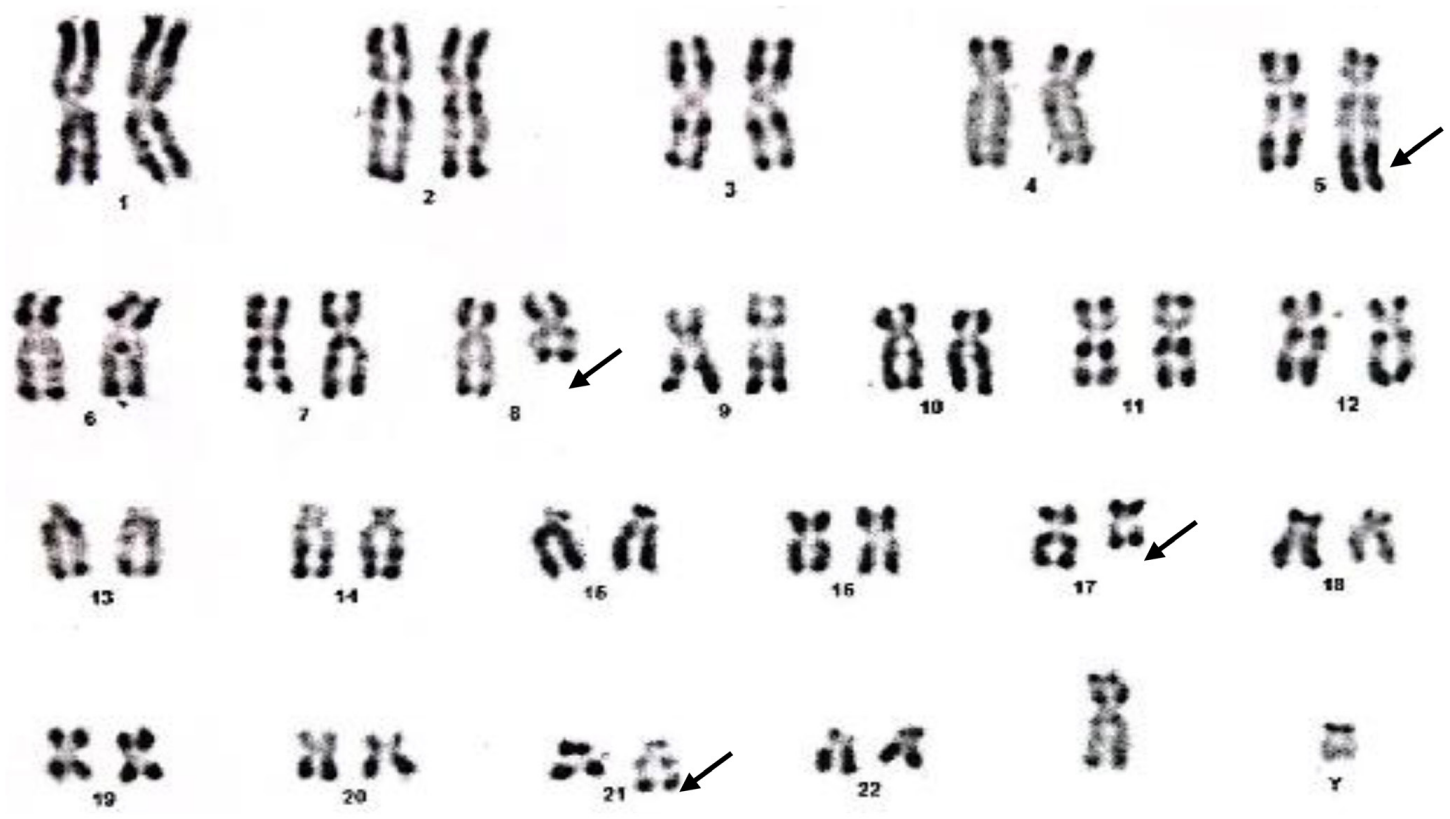
46, XY, t (8, 21) (q22; q22) with t (5, 17) (q35, q21) Karyotype.

A 19-year-old male patient presented in December 2017 with a one month history of fever and asthenia. Physical examination was normal; particularly absence of lymphadenopathy and splenomegaly. The white blood cell count was 42 000/mm^3^ consisting of 10% neutrophils, 10% lymphocytes, and 80% blasts. The hemoglobin was 6.9 g/dl and the platelet count was 10 000/mm^3^. Coagulation test showed disseminated intravascular coagulation (DIC) with a prolonged activated partial thromboplastin time of 45 sec, prolonged prothrombin time and an increased d-dimers concentration of 1460 mg/mmol. Bone marrow analysis revealed increased blasts (98%) with typical morphology of AML type 1 according to the FAB classification: The cytoplasm is moderately basophilic, sometimes containing fine granules and/or Auer bodies (Figures 1 and 2). Flow cytometric analysis showed that the leukemic cells were positive for CD13, CD33, CD117, CD34, and MPO. Cytogenetic study of the bone marrow cells revealed in all the AML cells a 46, XY, t (8, 21) (q22; q22) with t (5, 17) (q35, q21) [20 mitoses] Karyotype (Figure 3). RT-PCR analysis performed on the bone marrow aspirate revealed the presence of AML/ETO fusion transcript (Rearrangement of RUNX1-RUNX1T1). Due to a shortage of probe, the research of the rearrangement NPM1-RARA was not performed. The patient has reached cytologic and cytogenetic remission after undergoing induction therapy with cytarabine (200 mg/m²) for 7 days associated with idarubicin (12 mg/m²) for 3 days. The all-trans retinoic acid (ATRA) was not received and the DIC was managed by Fresh Frozen Plasma (FFP) transfusion. After that, the patient received two cycles of consolidations, the first associated cytarabine 200 mg/m² × 7 days, daunorubicin 60 mg/m^2^ × 3 days and etoposide 100 mg/m^2^ × 5 days and the second consisted of high dose cytarabine 6 g/m² for 3 days. The bone marrow evaluation revealed persistent cytologic and cytogenetic remission but a positive minimal residual disease (MRD) indicating the allogeneic transplantation of stem cells. RT-PCR for RUNX1- RUNX1T1 before the allograft was negative. The patient is so far alive in persistent complete remission.

## DISCUSSION

The t (8; 21), is reported to be found in 5–10% of all AML cases [5, 6]. On the morphological level, this entity is characterized by the presence of large blasts having basophilic cytoplasm associated with numerous azurophilic granules and a perinuclear clearing. Auer bodies are also commonly reported and may be detected in blasts or immature neutrophils. Bone marrow examination frequently reveals granulocytic maturation phases to promyelocytes, myelocytes and mature neutrophils that could sometimes be associated with dysplasia features [7]. The t (5; 17) in AML is exceptional, described in few cases in APL variant [3, 4]. The morphology usually shows a Hypergranular and hypogranular bilobed promyelocytes; absence of Auer rods; typical microspeckled pattern with anti-RARa antibodies [3, 4]. In our patient, this unusual association of t (5; 17) to t (8; 21) doesn’t affect the morphology of the cells and the bone marrow aspiration shows blasts with typical morphology of AML type1 according to the FAB classification: The cytoplasm is moderately basophilic, sometimes containing fine granules and/or Auer bodies.

On the immunological level of AML with t (8; 21), the cells, usually tend to express the following markers: high levels of CD34, HLA-DR, myeloperoxidase (MPO) and CD13 (7). A relative weak expression of CD33 was found, and aberrant lymphoid associated markers (CD19, CD56, cCD79a, Pax5) expression was reported with variable prognosis implications [8–10]. Moreover, on flow cytometry assessment in our case, we concluded to the expression of the following antigens CD13+, CD33, CMPO+, CD117+, CD34+ which are typically described with t (8; 21) and therefore, this unusual association of t (5, 17) to t (8; 21) doesn’t affect the immunological phenotype of the cells.

In few cases with a morphological diagnosis of APL, patients have variant chromosome translocations, which fuse RARA gene with partner genes other than PML, such as in the variant translocation t (5; 17) (q35; q21) that fuses the N-terminus of nucleophosmin (NPM1) gene at 5q35 to the retinoic acid receptor alpha at 17q21 [11, 12]. In our case, NPM1-RARA not realized for lack of the specific probe.

In APL with the classic reciprocal translocation t (15; 17) (q22; q21) which is characterized by fusion gene transcript PML-RAR-alpha, patients are responsive to differentiation treatment with all-trans retinoic acid (ATRA) [13]. In our case, the research of NPM1-RARA was not possibly due to scarcity of probes and therefore the patient didn’t receive the ATRA.

Unbalanced translocation der (5; 17) resulting in a TP53 loss [14], whole-arm translocation of der (5; 17)(p10; q10) with concurrent TP53 mutations [15] and reccuring abnormality dic (5; 17) associated also with mutations of TP53 [16] were described in AML. The association with P53 abnormalities was not tested in our patient.

Up to now only 7 cases with balanced t (5; 17) (q35; q12-21) translocation and the underlying NPM1/RARA fusion have been identified, remission obtained with chemotherapy associated or not with ATRA. The different cases were detailed in Table 1.

**Table 1:** Reported cases with t (5; 17)

CASE	Age (Year)/ Sex	Caryotye	DIC	Treatment	Outcome
Redner 1994 [3]	2/F	46, XX, **t (5; 17) (q32; q12)** [17]/ 46, XX [3]	−	Ara-C + DNR + 6MP + VP16 → ATRA + autoBMT	CR → relapse and died
Veronique 1995 [4]	76/F	45, XX, der(5) **t (5; 17) (q?; q12),** de1(8) (q22 q24), −17, 5–32 dmin [31]	NA	AraC+DNR+ATRA	Died, septicemia
Hummel 1999 [11]	12/M	47, XY, **t (5; 17) (q35; q21)** der(8)(p23), der(10) (q26), del(12) (q13 q22), del(1) (q12 q14), −16, −18, +21, +22, +mar	+	Ara-C + DNR + consolidation → AraC and ATRA + BMT	CR → relapse And died
Grimwade 2000 [12]	9/F	46, XX, ins(3; 5) (q26; q13 q13), **t (5; 17)** **(q34; q21)**	−	Ara-C + Mit + ATRA	Alive in CR
XU 2001 [17]	12/M	46, XY, del(12)(p13), **t (5; 17) (q35; q21)** [6]/46, XY [10]	+	ATRA days 1–5	Died from cerebral hemorrhage
Nicci 2005 [18]	29/M	46, XY, **t (5; 17) (q35; q21)** [23]/ 46, XY [4]	NA	ATRA, chemo → ATO	Relapse, Alive
Otsobou 2012 [19]	4/M	46, XY, **t (5;17) (q35; q12)**, i (21) (q10) [16]/ 46, XX [4]	+	ATRA + chemo	Alive in CR
Kikuma 2015 [20]	51/M	46, XY**, t (5;17) (q35; q12)** [14]/ 46, XY [2]	+	ATRA, chemo	Alive in CR
present case	17/M	46, XY; **t (5; 17) (q35; q21)**; **t (8; 21)** (q22; q22)	+	chemo (Ara-C + Ida) + BMT	Alive in CR

Abbreviations: M: male; F: female; DIC: disseminated intravascular coagulation; DNR: daunorubicin; Ara-Ccytarabine; 6MP: 6-mercaptopurine; VP16etoposide, Mitmitoxantrone, IdaIdaribicin; ATRA: all-trans retinoic acid,chemo chemotherapy; ATO: arsenic trioxide; BMT: Bone marrow transplantation; CR: complete remission; NA: not available.

In two studies [3, 11] (Table 1), the ATRA associated with chemotherapy was used in second line therapy leading to short remission in patients with t (5; 17) suggesting its beneficial effect in first line therapy. Chemotherapy with differentiation therapy has been used in the majority of cases of the t (5; 17) in the literature [12, 19, 20]. The use of differentiation therapy alone (ATRA, Arsenic trioxide) has not been reported. While patients’ response to ATRA alone is difficult to assess since chemotherapy was part of the induction treatment, the differentiation effects of ATRA probably led to remission in these patients. Indeed, Redner and colleagues have demonstrated that in short term culture systems, cells bearing the t (5; 17) translocation terminally differentiate in response to ATRA [21]. Our case is very interesting describing, to our knowledge, the first case of t (8; 21) associated with t (5; 17). Bone marrow examination revealed positive MRD by flow cytometry (positive at threshold 10^−4^) after consolidation which led to the decision to recommend allogenic stem cell transplantation. Our patient didn’t receive ATRA in combination with chemotherapy (Marketing Authorization in Tunisia for only APL) and perhaps if he received it, he would have negative residual disease after the consolidation and therefore no indication for allogenic stem cell transplantation. The prognosis is difficult to assess, and further accumulation of data on such cases is desirable in order to be able to conclude.

## CONCLUSION

We have described a patient with a novel balanced t (5; 17) (q35; q21) translocation emerging with t (8; 21) in AML. To our knowledge, this additional chromosomal aberration has not yet been described. This aberration is thought to play a crucial role in prognosis. The efficacy of ATRA is controversial. Therefore, further accumulation of data on such cases is warranted to enable more comprehensive and conclusive evaluations.
